# Co-circulation and co-infection of COVID-19 and influenza in China: challenges and implications

**DOI:** 10.3389/fpubh.2023.1295877

**Published:** 2023-12-08

**Authors:** Qingchun Pan, Zhenghao Tang, Yongsheng Yu, Guoqing Zang, Xiaohua Chen

**Affiliations:** Department of Infectious Diseases, Shanghai Sixth People's Hospital Affiliated to Shanghai Jiao Tong University School of Medicine, Shanghai, China

**Keywords:** COVID-19, SARS-CoV-2, influenza, co-circulation, co-infection, public health, vaccination

## Introduction

**Figure 1:**
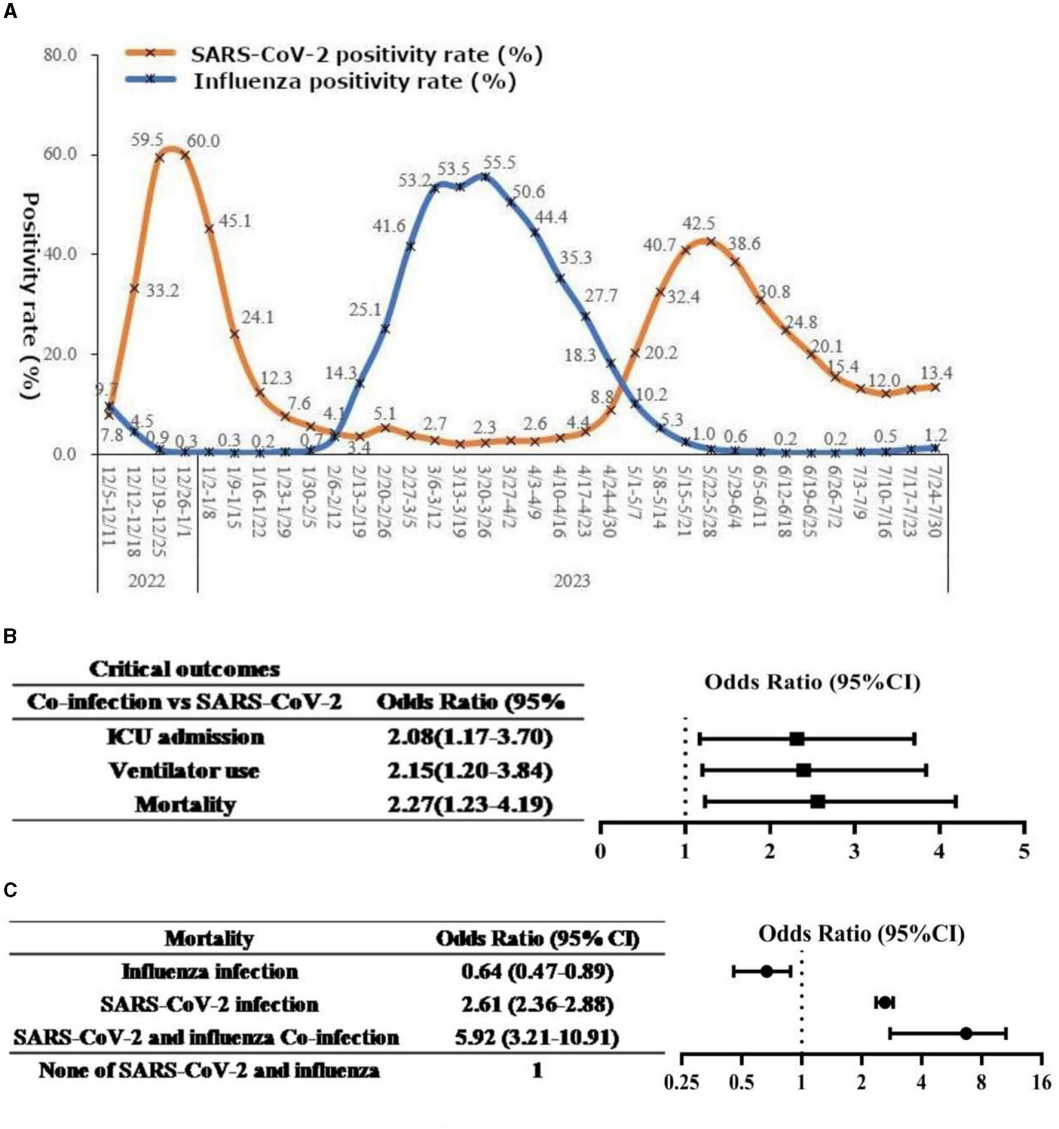
**(A)** National SARS-CoV-2 and influenza virus infection situation in China. Sourced from: Chinese Center for Disease Control and Prevention (1). **(B, C)** Forest plots of the association between co-infection and disease outcomes. Example chart presenting data from: Stowe et al. (2). **(B)** Forest plot of the association between co-infection and critical outcomes. **(C)** Forest plot of the association between co-infection and mortality.

The global landscape has been profoundly shaped by the SARS-CoV-2 pandemic, and China also faces significant challenges. Unlike most countries in the world that have adopted an open approach, China ended its “Zero COVID” policy (refers to a set of measures taken by governments with the aim of reducing the number of COVID-19 cases to zero. It typically includes strict lockdowns, early testing, contact tracing, and other measures to quickly control the spread of the virus and eliminate the outbreak) on December 7, 2022. The sudden termination of this policy resulted in two SARS-CoV-2 outbreaks within a six-month period, accompanied by an unexpected increase in influenza cases (Figure 1A) (3). This sequential rather than simultaneous exposure of the population to COVID-19 and influenza, resulting in successive acquisition of corresponding immunity. The fluctuating outbreaks of COVID-19 and influenza, coupled with the high variability of both viruses, increased susceptibility to new variants within the population. Simultaneously, the seasonal patterns of these two respiratory infectious diseases persisted, raising the risk of co-circulation and giving rise to a range of distinct challenges. Compared to single-virus infections, individuals co-infected with COVID-19 and influenza experienced greater disease severity (2, 4, 5). Considering the disproportionate burden borne by susceptible populations in terms of COVID-19 and influenza-related illnesses, coupled with the existence of a substantial vulnerable population in China, this heightened severity is particularly concerning. As immunity wanes, especially among vulnerable groups, coupled with the relaxation of public health and social measures, the co-circulation and co-infection of COVID-19 and influenza in the upcoming seasonal outbreak pose a novel, potential health threat. While the probability remains low (6), the concurrent circulation and infection of these two diseases could also pose significant challenges to China's healthcare system. Therefore, it is imperative to address this issue in China. This commentary aims to emphasize the critical importance of focused efforts to understand and effectively manage the intricate dynamics of COVID-19 and influenza co-circulation and co-infection. By doing so, we can better prepare for and mitigate the potential challenges that lie ahead.

## Seasonal patterns of influenza and COVID-19

Understanding the seasonal dynamics of influenza and COVID-19 is pivotal for effective control strategies. Before the COVID-19 pandemic, influenza showed clear seasonal patterns in mainland China based on latitude. Influenza A typically had a winter epidemic in Northern provinces above 33°N, peaking in January or February. In the southernmost provinces below 27°N, a single peak occurred every April to June, while mid-latitude regions experienced dual-peak epidemics in January to February and June to August (7). However, the implementation of stringent measures during 2020–2022 disrupted these patterns due to the zero-COVID-19 policy. Mainland China's zero-COVID-19 policy and strict non-pharmaceutical interventions (NPIs are actions, apart from getting vaccinated and taking medicine, that people and communities can take to help slow the spread of illnesses) significantly reduced influenza cases and altered the typical seasonality in 2020–2022. Yet, this situation changed in 2023, with the policy's end and relaxed restrictions, Influenza A made a resurgence at the end of February, more severe than historical outbreaks (8). Notably, these atypical outbreaks occurred at different times compared to pre-pandemic influenza patterns, delayed by about 2–3 months from north to south. Consequently, timely implementation of recommended prevention measures for vulnerable populations was disrupted, thereby influencing public health decisions. Hence, comprehending the evolving interplay between influenza and COVID-19 seasonality, particularly against the backdrop of changing policies, is of paramount importance for effective joint control strategies.

## Monitoring and data collection

Effective public health response relies on meticulous monitoring of influenza and COVID-19 activity. The Chinese National Influenza Center (CNIC) and the China Disease Prevention and Control Information System (CDCIS) play pivotal roles in the nationwide surveillance. Leveraging advanced methodologies and technologies, these institutions compile comprehensive epidemiological data on influenza and COVID-19. Their regular reports provide a breakdown of virus strains detected by type and subtype, offering critical insights into the nationwide trends (3, 8). Given the novel nature of COVID-19 and its historical interaction with NPIs, the precise seasonal pattern of the virus remains under exploration. Limited research, however, suggests potential similarities in seasonal behavior between COVID-19 and influenza (9). This raises the concern of co-circulation during the influenza season, thus amplifying the risk of concurrent COVID-19 and influenza infections. Notably, co-infection with influenza A virus could exacerbate the infectivity of SARS-CoV-2 and intensify the severity of COVID-19 (10), further underscoring the impact of such co-infections on affected populations. To date, monitoring and data collection remain crucial in unraveling the complex dynamics of co-circulating viruses and their implications for public health strategies.

## Challenges and consequences of co-circulation and co-infection

Currently, there is limited global reporting on co-infections of COVID-19 and influenza. It is worth noting that China's strict NPIs in the past 3 years effectively reduced the possibility of simultaneous infection with SARS-CoV-2 and influenza viruses. However, following the termination of zero COVID-19 policies, staggered outbreaks of COVID-19 and influenza have led to temporary immunity against either disease for many individuals (11). As time progresses, this pattern of staggered outbreaks may disappear. It is expected that COVID-19 and influenza will circulate jointly, posing new health challenges to the ever-growing vulnerable population—an aging population and the growing prevalence of chronic diseases in China. The inherent variability of influenza and SARS-CoV-2 further complicates the situation. Immunity acquired through infection or vaccination may be evaded by continuously evolving variants, thereby increasing susceptibility to new strains within the population. This also creates greater opportunities for the re-emergence of both viruses during future influenza seasons. With the increased local and international travel, the risks of introducing and spreading influenza viruses and new variants of SARS-CoV-2 are further amplified. While early co-infections were primarily associated with non-Omicron variants of SARS-CoV-2, the widespread presence of the Omicron variant exhibits heightened transmissibility, raising concerns (12). Recent research conducted in Brazil revealed higher fatality rates in cases of concurrent infections involving the Omicron variant of SARS-CoV-2 and influenza A (H3N2) compared to single infections (13). These findings underscore the serious consequences of co-infections and provide insight into the potential impacts of upcoming seasonal outbreaks. It is crucial to proactively consider these challenges in order to improve public health strategies and effectively address the complex dynamics of the joint circulation of these two viruses in the evolving situation.

## Clinical outcomes of co-infection

Co-infection of COVID-19 and influenza has been shown to result in more severe clinical outcomes, such as mortality, mechanical ventilation, and ICU admission (2, 4, 5). While there is no significant difference in the severity of clinical symptoms, such as fever, cough, and laboratory indices, including white blood cells, C-reactive protein, and inflammatory cytokines, between co-infection and single infection (14), animal experiments have demonstrated that co-infection leads to more severe lung damage than single infection with SARS-CoV-2 alone (15). Additionally, studies have shown that compared to single infection with SARS-CoV-2, patients with co-infection have a higher hospitalization rate (85.7 vs. 6.7%) and are more likely to develop complications such as acute hypoxic respiratory failure, acute respiratory distress syndrome, cardiac injury, and acute kidney injury (16–18). In cases of co-infection, key clinical outcomes, including mortality rates, mechanical ventilation rates, and ICU admission rates, are also elevated (Figure 1B), with a specific focus on the higher mortality rate, as illustrated in Figure 1C, particularly among vulnerable populations such as the older adult (2). Both SARS-CoV-2 and influenza can cause respiratory complications, as well as non-respiratory complications involving various organ systems (19). COVID-19 patients face a higher risk than influenza patients. However, there is currently limited information on whether co-infection with influenza increases the risk of non-respiratory complications in COVID-19 patients, which requires further observational research. Moreover, it is worth exploring whether co-infection with influenza or reinfection with influenza during the duration of Long COVID, which is broadly defined as signs, symptoms, and conditions that continue or develop after acute COVID-19 infection, can worsen or prolong the symptoms of Long COVID. This aspect of co-infection's impact on Long COVID-related symptoms merits further investigation.

## Disease burden of co-infection

Co-infection of COVID-19 and influenza may lead to a substantial disease burden. In China, there are annually 88,000 excess deaths attributed to influenza-related respiratory illnesses, with individuals aged 60 and above comprising 80% of the total mortality (7). Since the cessation of the “Zero COVID” policy, China has reported over 80,000 COVID-19-related deaths. Both influenza and COVID-19 impose significant economic burdens, with China's economic burden due to influenza-related diseases reaching ¥26.381 billion in 2019 (7). Compared to influenza, COVID-19 presents an increased risk of complications and mortality, longer hospital stays, and higher medical costs (19). Among individuals infected with COVID-19, those aged 65 and above exhibit the highest hospitalization rate (43.06%) and mortality rate (76.07%) (20). Official reports regarding the disease burden of COVID-19 in China have not been publicly disclosed. Referencing a study from the United States, co-infected individuals had higher mean hospitalization costs (USD 129,742 vs. USD 68,878, *p* = 0.04) and longer total length of stay (9.9 days vs. 8.2 days, *p* = 0.01), a higher likelihood of requiring mechanical ventilation (OR 2.01, 95% CI 1.19–3.39), and a higher in-hospital mortality rate (OR 2.09, 95% CI 1.03–4.24) compared to those with sole COVID-19 infection (21). Considering China's larger population, an increasing older adult population, and a growing number of individuals with chronic diseases, there exists a substantial population of vulnerable individuals. Furthermore, China's current healthcare system is less advanced than that of the United States. Given the significant disease burden caused by COVID-19 in the United States and the worse clinical outcomes associated with co-infections compared to single infections, the disease burden resulting from co-infections may be even more severe and challenging to estimate in China. Therefore, heightened awareness of the threat of co-infections, especially among vulnerable populations, is of paramount importance.

## Low vaccination rates and importance of vaccination

Prior to the end of the zero-COVID-19 policy, China aggressively promoted the COVID-19 vaccine for all individuals, with official data indicating a vaccination rate of 90.5% for the general population and 96% for those aged 60 or above (1). The majority of individuals have achieved hybrid immunity in the wake of recent COVID-19 outbreaks. Nevertheless, now COVID-19 vaccination is no longer a topic of discussion. Despite the decline of immunity and the approaching season when COVID-19 may resurge, there appears to be a lack of concern about COVID-19 vaccination among the population. Meanwhile, the long-term influenza vaccine uptake rate in China remains low (<5%), far below that of developed countries (~50%). In comparison to the general population, vulnerable populations in China have similarly low vaccination rates for both COVID-19 and influenza vaccines, particularly the latter. The influenza vaccination uptake rate among individuals aged 60 or above is 3.8%, while that among individuals with chronic diseases is 4.0% (22). Hesitancy among vulnerable populations regarding vaccine uptake is driven by factors such as concerns about vaccine side effects and efficacy, a belief that they are at low risk of infection, and self-determined contraindications, such as individuals with well-controlled chronic conditions (e.g., hypertension, diabetes, chronic liver and kidney diseases), those in a stable period of cancer, and those who self-identify as allergy-prone. Despite the well-known benefits of vaccination in preventing corresponding diseases, studies have surprisingly demonstrated that influenza vaccination can reduce the incidence and severity of COVID-19 infections (23, 24), and decrease the mortality attributable to co-infection of influenza and SARS-CoV-2 (25). These findings underscore the importance of vaccine uptake, particularly for public health and the health of vulnerable individuals in the post-COVID-19 era.

## Discussion and recommendations

Although co-infections may not occur frequently, given the large population in China and the unequal access to healthcare, it is challenging to accurately predict the quantity and severity of future co-infections. From the perspective of public health and clinical care, the burden on the healthcare system caused by the prevalence of a single virus is already significant, not to mention the burden of overlapping epidemics, which has become a credible threat. Therefore, even a relatively low co-infection rate in China could impose a heavy disease burden on healthcare systems, especially when considering relaxed public health and social measures. In conclusion, despite the end of the nationwide wave of SARS-CoV-2 and the approaching end of the influenza season, both viruses continue to circulate and may lead to overlapping epidemics in the next season, resulting in an increase in co-infections. Therefore, the threat posed by co-infections to population health persists and remains a Damocles sword hanging over the Chinese healthcare system. Preventing infection, strengthening surveillance, and actively promoting vaccine uptake are still the best approaches to address this threat.

## Author contributions

QP: Conceptualization, Visualization, Writing—original draft. ZT: Data curation, Writing—original draft. YY: Data curation, Writing—original draft. GZ: Supervision, Writing—review & editing. XC: Resources, Writing—review & editing.
